# Theory-based behavioral change interventions to improve periodontal health

**DOI:** 10.3389/froh.2023.1067092

**Published:** 2023-01-25

**Authors:** Charlotte C. K. Chan, Alice K.Y. Chan, C.H. Chu, Y. C. Tsang

**Affiliations:** Faculty of Dentistry, The University of Hong Kong, Hong Kong, Hong Kong SAR, China

**Keywords:** behavioral change, behavioral intervention, periodontal health, oral hygiene instruction, preventive dentistry

## Abstract

Periodontal disease is a significant global health burden affecting half of the world's population. Given that plaque and inflammation control are essential to the attainment of periodontal health, recent trends in preventive dentistry have focused on the use of behavioral models to understand patient psychology and promote self-care and treatment compliance. In addition to their uses in classifying, explaining and predicting oral hygiene practices, behavioral models have been adopted in the design of oral hygiene interventions from individual to population levels. Despite the growing focus on behavioral modification in dentistry, the currently available evidence in the field of periodontology is scarce, and interventions have primarily measured changes in patient beliefs or performance in oral hygiene behaviors. Few studies have measured their impact on clinical outcomes, such as plaque levels, gingival bleeding and periodontal pocket reduction, which serve as indicators of the patient's disease status and quality of oral self-care. The present narrative review aims to summarize selected literature on the use of behavioral models to improve periodontal outcomes. A search was performed on existing behavioral models used to guide dental interventions to identify their use in interventions measuring periodontal parameters. The main models were identified and subsequently grouped by their underlying theoretical area of focus: patient beliefs (health belief model and cognitive behavioral principles); stages of readiness to change (precaution adoption process model and transtheoretical model); planning behavioral change (health action process approach model, theory of planned behavior and client self-care commitment model); and self-monitoring (self-regulation theory). Key constructs of each model and the findings of associated interventions were described. The COM-B model, a newer behavioral change system that has been increasingly used to guide interventions and policy changes, is discussed with reference to its use in oral health settings. Within the limitations of the available evidence, interventions addressing patient beliefs, motivation, intention and self-regulation could lead to improved outcomes in periodontal health. Direct comparisons between interventions could not be made due to differences in protocol design, research populations and follow-up periods. The conclusions of this review assist clinicians with implementing psychological interventions for oral hygiene promotion and highlight the need for additional studies on the clinical effects of behavioral model-based interventions.

## Introduction

Periodontal conditions affect a significant proportion of the world's population, with severe periodontitis affecting 1 in 7 globally (1, 2). Despite recent scientific and technological advances that have transformed dental care in the clinical setting, the maintenance of oral health remains dependent on the patient's daily habits at home (3). With the shifting focus of dental care to that of disease prevention, the latest European Federation of Periodontology (EFP) clinical practice guidelines recommend dentists to motivate their patients to achieve adequate oral hygiene practice in order to prevent periodontitis onset and progression (4). Dentists are advised to implement motivation and behavioral change on patients in step one therapy before proceeding to step two and step three therapy to improve treatment compliance and response according to the latest clinical guidelines for treatment of stage I to stage III periodontitis patients.

While much of traditional oral hygiene education involves telling the patient what to do, why one should engage in such behaviors, and how they should be performed, this one-sided transfer of information often fails to consider the patient's perspective. Reviews of dental education programs found that most interventions achieved short-term improvements in oral health knowledge but failed to produce long-term behavior changes and clinical improvements in periodontal health (5). Rather than assuming that a knowledge deficit is the only reason for poor oral habits, one must delve deeper into the underlying factors that shape patient adherence. Despite the increased provision of public education programs promoting a balanced diet and exercise, leading to higher societal awareness of their importance, obesity rates are still on the rise (6, 7).

Studies have yet to identify an association between knowledge of nutritional guidelines and actual consumption of the recommended foods (8, 9). Similarly, given the abundance of available information in the media and on the Internet, knowledge alone is unlikely to be sufficient in causing oral hygiene behavioral change. Other determinants of behavioral change must be explored by the dentist and be addressed appropriately. Whether a patient competently performs desirable oral hygiene habits is multifactorial. Those factors can be classified as personal factors such as motivation, beliefs, and intention, and external factors such as access to appropriate tools, a conducive physical environment, and social norms and expectations (10).

To understand the components of behavioral change, various models in the field of psychology have been proposed which have been applied and studied in settings ranging from smoking cessation to dietary change (11, 12). In recent years, oral hygiene interventions (OHI) incorporating behavioral change models have been tested (13). Systematic reviews reported tentative evidence that psychological interventions can improve oral hygiene (3, 14–16). At the 11th European Workshop on the prevention of periodontal and peri-implant diseases, experts recommended the use of psychological approaches to improve plaque control in periodontal management (17).

While there is evidence to support the use of interventions to increase the frequency of oral hygiene behaviors, the clinical implications of this increased performance are less clear (18). In clinical periodontal practice, bleeding on probing and probing depth provide direct information about gingival inflammation and periodontal disease severity, influencing diagnosis and management and hence both are considered important parameters to be monitored (19). This article therefore aims to explore the outcomes of theory-based behavioral change interventions through periodontal health and hygiene indicators, such as gingival bleeding, attachment loss and plaque scores. A review of the current literature was done to examine the clinical impact of behavioral change interventions, which were traced back to their underlying psychological theory. To highlight the key concepts of behavior change theory and assist clinicians in planning interventions, the following discussion of traditional models is grouped by their common theoretical area of focus: patient beliefs, readiness to change, planning, and self-monitoring. The former two groups relate to understanding the nature of behavior to be changed, while the latter two relate to the techniques with which such change can be achieved. A more recent behavioral change model, COM-B, is also explored. The main constructs of each psychological approach are described, and the findings of the associated interventions presented.

The objective of this study is to give an overview of the key concepts in behavioral change theory-based interventions and their usefulness in clinical care, ultimately facilitating the future delivery of effective oral hygiene instruction to improve patient motivation, compliance and adherence.

## Interventions based on patient beliefs

### Health belief model

The Health Belief Model (HBM) proposed by Rosenstock in 1966 is one of the earliest theories of health behavior and amongst the most widely used (20). The author suggested that behavior is influenced by beliefs about the risk of developing a health problem (“perceived susceptibility”), the extent to which it would affect the individual (“perceived severity”), the value of performing the behavior (“perceived benefits”) and the obstacles to doing so (“perceived barriers”). The final two constructs are “self-efficacy”, the belief that one can successfully perform the required behavior, and “cues to action”, which are circumstances or events that trigger the individual to become ready for behavioral change—such as noticing bleeding gums or halitosis (21, 22).

**Figure 1:**
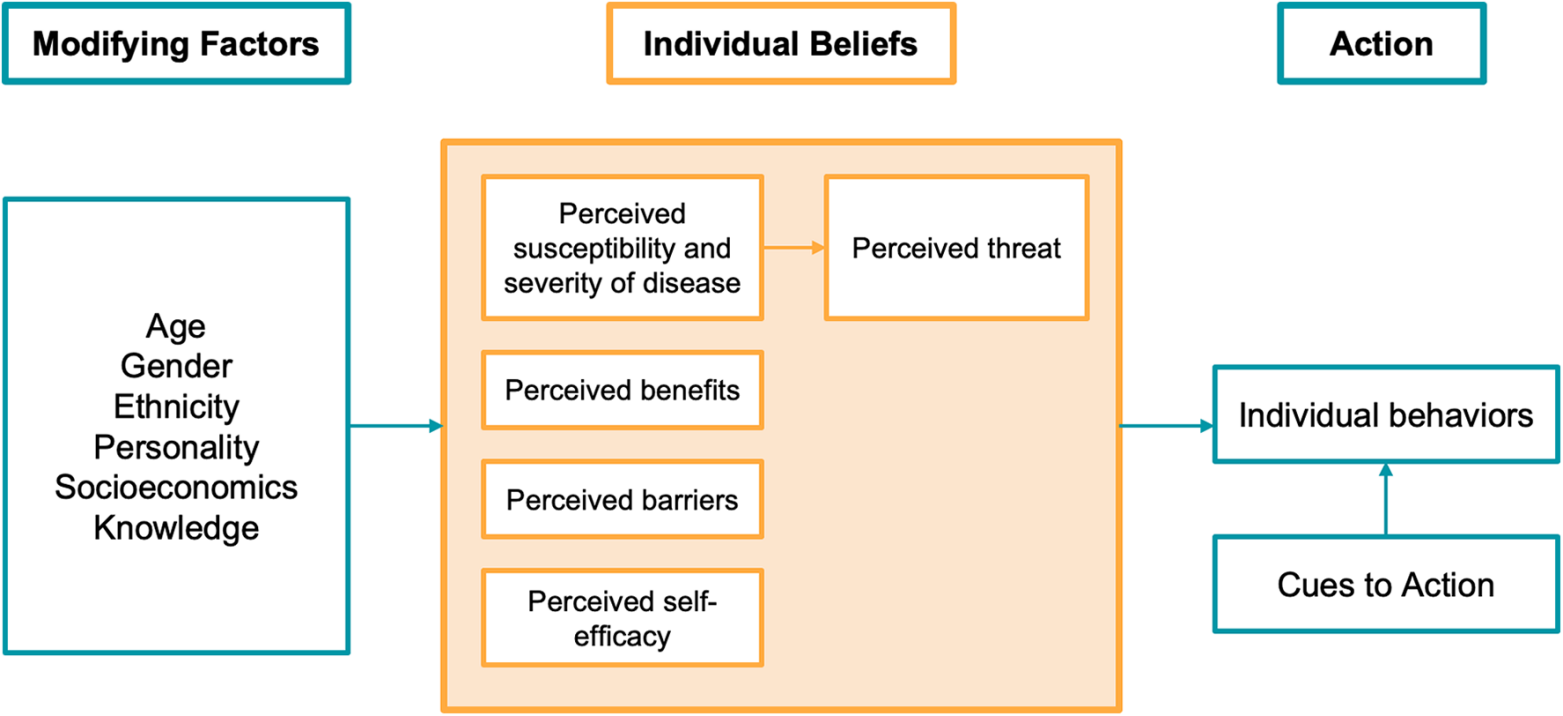
Health belief model components and linkages (22).

Figure 1 shows the components of The Health Belief Model. According to the model, for patients to act in response to oral hygiene instruction, they would need to believe that they are at high risk for periodontitis and that the associated consequences of tooth loss and systemic disease are severe. If they believe that they are capable of flossing and brushing and that the benefits of remaining periodontally healthy outweigh the time and effort required to perform such actions, then they are more likely to change their behavior.

In the past decade, multiple randomized controlled trials of OHI based on the HBM have been conducted. Jeihooni, Jamshidi (23) noted a significant increase in the constructs of HBM and self-reported oral hygiene practices in the experimental group, suggesting that HBM can be applied to predict behavior in the dental context. Higher self-efficacy is correlated with higher levels of brushing and flossing and lower levels of bleeding on probing (24). A meta-analysis concluded that interventions based on HBM are effective in improving oral health behaviors (14).

As for clinical outcomes, a study involving 34 Chinese adults with periodontitis found that those who received an additional HBM intervention had significantly lower full mouth plaque and bleeding scores than those who received oral hygiene instruction alone (25). Similarly, Rivandi, Garmaroudi (26) found that adults with periodontitis and gingivitis who received the HBM-based OHI not only experienced significant changes in HBM constructs compared to the control but also presented with reduced pocket depth and plaque index. However, as the control group received only root planing, it is unclear whether the improvements were due to HBM-specific education, or the general delivery of education itself.

Given the importance of establishing good oral habits from a young age, OHIs have frequently been used in child and adolescent populations. In a randomized controlled trial involving 1,159 adolescents in Hong Kong, those receiving OHI based on the HBM had a statistically significant decrease in visible plaque index after 12 months, compared to the control group who received informational booklets on oral hygiene (27). Improvement in oral hygiene, measured by plaque score (28, 29) and simplified oral hygiene index (30), was also found in three randomized controlled trials. Two other randomized controlled trials involving school-aged children found significant improvements in gingival inflammation, reflected by the papillary bleeding index (31) and bleeding on probing (28).

### Cognitive behavioral theory

Although not strictly a model, cognitive behavioral theory (CBT) has been included in reviews of psychological approaches used to guide behavioral change interventions with promising results (3, 15, 32). Like the HBM, CBT also explores how perceptions and beliefs influence individual actions. Initially used for the management of dental anxiety, CBT has gained popularity in the field of behavioral change interventions due to its widespread success and cost-effectiveness (33). It aims to make patients aware of the interconnectedness of one's thoughts, feelings and actions and suggests that change in the latter can be achieved by influencing the former. One of the ways in which CBT principles are applied to OHI is through motivational interviewing, where the dentist collaboratively and openly explores with patients their feelings, beliefs and perspectives (34).

Schensul, Salvi (35) investigated whether addressing cognitive mediators could impact behavior and clinical outcomes and found that patients expressing greater intentionality and locus of control to maintain oral health had greater brushing and flossing behavior and presented with lower gingival index scores, while those with negative beliefs (e.g., worries about self-management of oral hygiene) and emotions (e.g., fears of oral diseases) had higher plaque scores. A CBT intervention used in patients undergoing periodontal surgery found that compared to those who received the surgery alone, CBT led to a reduction in irrational beliefs and expectations, resulting in reduced distress, anxiety and pain (36). A more positive dental experience may improve patient compliance with self-care and supportive maintenance therapy, resulting in better treatment outcomes. Six randomized controlled trials of OHIs based on principles of CBT with follow-up periods from 2 to 6 months also demonstrated clinically evident improvements in periodontal conditions in terms of reduced probing depths, lower plaque scores and lower bleeding indices in the study populations, which included both adults and adolescents, treated and untreated periodontitis patients (34, 37–41). These studies suggest that CBT can be a useful intervention to improve clinical parameters in periodontal health by influencing cognitive variables such as self-efficacy, which is also a construct of the HBM.

## Interventions based on readiness to change

### Stages of change and transtheoretical model

The Stages of Change Model (SCM), also known as the Transtheoretical Model, was proposed by Prochaska and DiClemente (42). It views behavior change as a process, rather than a single outcome. The first stage is “precontemplation”, in which the patient has no intention to act in the foreseeable future. The “contemplation” stage is marked by the patient's awareness that a problem exists and an intention to act within the next 6 months. The “preparation” stage is characterized by a readiness to act within the next 30 days, for example, buying a toothbrush without having started brushing. The final two stages involve “action”, defined as a changed behavior observed for less than 6 months, and “maintenance”, where the adopted behavior has been sustained for at least 6 months and the individual, with the support of the dentist, works to prevent relapse.

Better oral hygiene has been found in individuals at more advanced stages of change in the SCM, with decreases in the plaque and gingival indices corresponding with the progression to a higher stage of change (43). The SCM can also be applied to periodontitis patients to assess treatment compliance; patients in the maintenance phase of the model were more likely to attend appointments, with the compliance rate decreasing for each of the model's earlier stages of readiness (44).

Motivational interviewing (MI) can be used to assist in the determination of the patient's stage of readiness in the SCM and identify progression over time in order to plan the appropriate oral health education. MI respects the patient's autonomy and individuality, acknowledging that those who are not ready to change are less likely to respond favorably to oral hygiene instruction, and has been found to be effective in improving clinical periodontal parameters in systematic reviews (45, 46).

### Precaution adoption process model

The Precaution Adoption Process Model (PAPM) from Weinstein is another model that describes the process of behavioral change in a series of stages. Compared to the SCM, the PAPM provides additional insight into patient's level of readiness as it differentiates seven stages of behavioral change: (1) unawareness of the importance of the behavior, (2) aware but unengaged, (3) accepts the issue and deciding about acting, (4) accepts the issue but decides not to change the behavior, (5) accepts the issue and decides to act, (6) takes action to change behavior, (7) maintenance (47).

**Figure 2:**
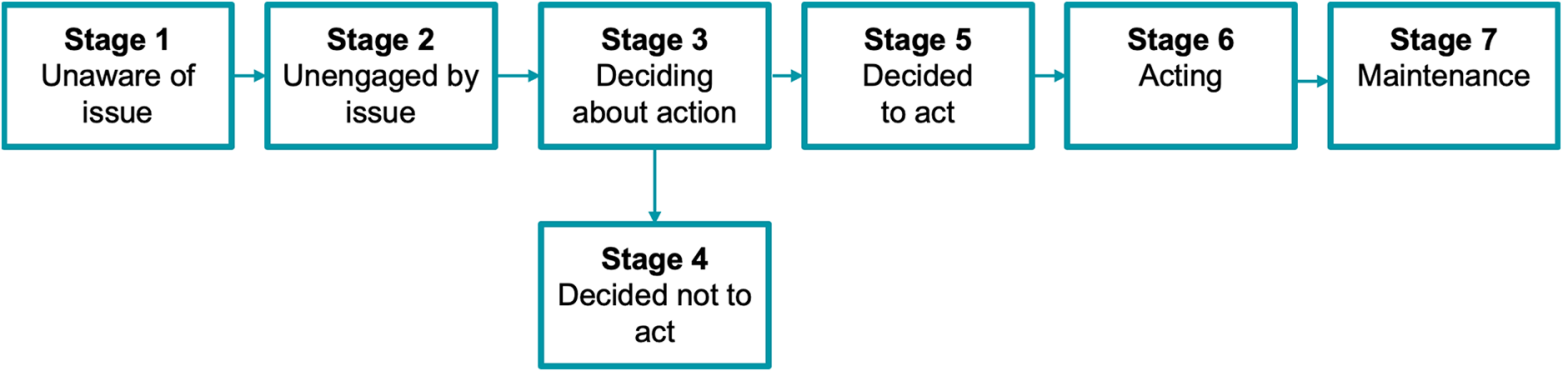
Stages of the precaution adoption process model (adapted from Weinstein and Sandman, 1992).

In a randomized controlled trial of 244 adolescents, subjects in the test group were identified based their stage of readiness according to the PAPM (48). Those assigned at Stages 1–4 received individualized OHI, consisting of basic knowledge on the etiology and prevention of oral disease, while those assigned at Stage 5 or higher of the PAPM received individualized instruction to carry out oral hygiene skills. After a follow-up period of 12 months, it was determined that the theory-guided OHI produced significant improvements in oral hygiene, assessed *via* plaque disclosing agent, compared to the control. Figure 2 illustrates the stages of the Precaution Adoption Process Model.

## Interventions based on planning behavioral change

### Theory of planned behavior

**Figure 3:**
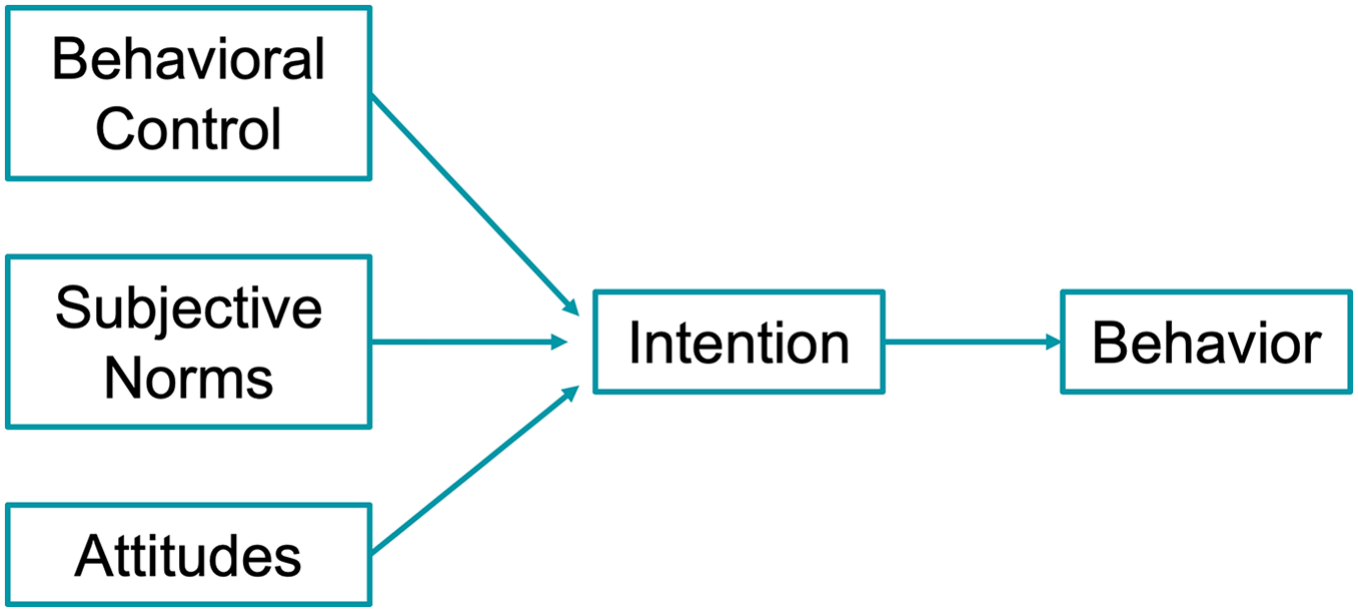
Theory of planned behavior [adapted from Hayden (50)].

While the SCM categorizes its preliminary stages based on an individual's level of intention to act, the Theory of Planned Behavior (TPB) concentrates on the intention itself. The theory, proposed by Ajzen (49) posits that intention is the main driver of behavior—in other words, those with a plan or aim to carry out the behavior are more likely to act. In the TPB, intention is shaped by attitudes, subjective norms and behavioral control. Like the HBM, the TPB considers how beliefs shape attitudes and the subsequent value placed on the behavior. However, while the HBM focuses on the individual, the TPB includes subjective norms in predicting the likelihood of behavioral change, defined as the “perceived social pressure to engage in a certain behavior” (50)—for example, social stigma associated with neglecting oral care, or family support to comply with dental treatment. The final component of intention is the perceived control over an individual's performance of the behavior, shaped by factors affecting the ease and difficulty of its execution. Figure 3 shows the Theory of Planned Behavior.

An OHI based on the TPB, in which the intervention group received didactic teaching and individual discussion on TPB constructs, resulted in significantly increased perceived behavioral control, flossing behavior, and reduced bleeding on probing (51). Other literature has shown that despite moderate success in using the TPB to predict intentions, an “intention-behavior gap” prevails in which the formation of intentions does not result in action (21). This led to the development of TPB-based interventions that focused on helping individuals plan how to turn their intentions into actual behavior. Sniehotta, Araujo Soares (52) devised an OHI measuring TPB variables that involved participants planning where, when and how they would floss. Those who participated in the planning exercise showed higher flossing compliance at 2-month follow-up. A study of 983 adolescents found that the TPB model explained 76.9% of the variance in dental brushing, measured by self-reported questionnaires and validated with visual plaque index (53). Perceived control became more predictive of actual behavior when action planning (i.e., when, where, how, how often and for how long to brush) and coping planning (i.e., what to do if the original plans are disrupted) were high. This supports the addition of elements of planning behavioral change to increase the effectiveness of OHIs, which has been demonstrated in previous studies (54–56).

### Health action process approach model

To address the limitations of the TPB, the Health Action Process Approach (HAPA) model was developed to include the role of planning in transforming intention into subsequent behavioral change. The HAPA consists of a goal-setting (“motivation”) phase and a goal-pursuit (“volition”) phase (57). A meta-analysis showed that action and coping planning, both psychosocial components of the volition phase in the HAPA model, are determinants of oral health behavior (58).

Four randomized controlled trials involving adolescents (59–62) investigated the effectiveness of HAPA theory-based OHIs involving action and coping planning. All four studies reported significantly improved periodontal conditions in the groups receiving the planning intervention compared to the controls, measured by reduced plaque (59–62) and community periodontal index scores (59, 62). In 2 studies, participants were asked to set oral health behavior goals, specifying when and where they would perform the behavior (action planning) (59, 60). When goals were not met, they were asked to create plans for how to deal with difficult or unexpected situations (coping planning), assisted by the provision of “volition sheets” describing commonly encountered difficulties in maintaining behavior change and solutions to overcome them. Those who formulated “if-then” plans to cope with potential circumstances that would jeopardize behavioral change or its maintenance experienced greater improvement in self-reported brushing and periodontal health, compared to those who only made action plans (62). This suggests that in addition to setting goals with an action plan, it may be worthwhile for the dentist to engage in coping planning with the patient through the discussion of contingency plans.

## Interventions based on self-monitoring

### Self-regulation theory

In addition to the setting of goals through action planning, OHIs have frequently incorporated self-monitoring to facilitate the patient's assessment of their own behavior in relation to their goals (15). As the dental practitioner is unable to physically supervise each instance of patients’ oral self-care, they must find ways to evaluate their own performances. Self-monitoring has been included in the behavioral change approach “GPS”, standing for goal-setting, planning and self-monitoring. GPS was deemed the most effective OHI to promote behavioral change in a systematic review and was recommended in the 11th European Workshop on Periodontology (15, 17).

Self-regulation theory, which has been defined as the ability to “plan, monitor and direct behavior in changing situations” (63), involves three interrelated activities: self-monitoring, self-evaluation, and self-reactions (64). Whereas the earlier described models included an action planning stage, self-monitoring forms part of action control and is a prerequisite for self-evaluation and self-reaction to occur (65). In dentistry, self-monitoring of oral hygiene behavior can be accomplished by using checklists, diaries or note-taking tools to record one's actions. The difficulties encountered when attempting the behavior can also be recorded. Self-monitoring can also be used to record the quality of the behavioral action, achieved by visual inspection with or without the use of disclosing agents, or personal experiences of clinical symptoms such as bleeding gums. By directing one's attention to the health problem, the patient gains a sense of agency and control over the behavioral change, which may encourage him to set more challenging goals (66).

Self-evaluation involves comparing the initial goal to the current state, as observed by self-monitoring. Self-reaction consists of an emotional response depending on the extent to which the goal has been achieved, as well as “self-efficacy expectations”, which result in judgments about future capability to perform the required behavior and achieve goals (66). For example, the observation that flossing and brushing behavior has led to an improvement in oral hygiene, as demonstrated by the plaque disclosing agent, may positively reinforce the behavior by affecting the individual's beliefs and motivation.

Randomized controlled trials have demonstrated that self-monitoring of oral hygiene behaviors in patients leads to improved oral hygiene through improved self-care habits (65, 67), lower bleeding on probing (38) and lower plaque indices (68, 69). Little, Hollis (70) conducted an OHI on 107 adults with moderate periodontal disease and found that the intervention group, who performed goal-setting and self-monitoring with calendars, had significantly greater flossing and brushing frequency and a significant reduction in full mouth plaque score, gingival bleeding, bleeding on probing, and periodontal pocket depth. Interestingly, Suresh, Jones (71) found that adults allocated to a self-monitoring intervention to improve flossing behavior presented with reduced plaque and bleeding scores regardless of their behavioral stage of change, suggesting that this technique could be used even in patients who were deemed not ready to change. However, these results were contradicted by Schuz, Sniehotta (72) where only those in the volitional phase benefitted from the self-monitoring intervention.

## Capability opportunity motivation-behavior model

**Figure 4:**
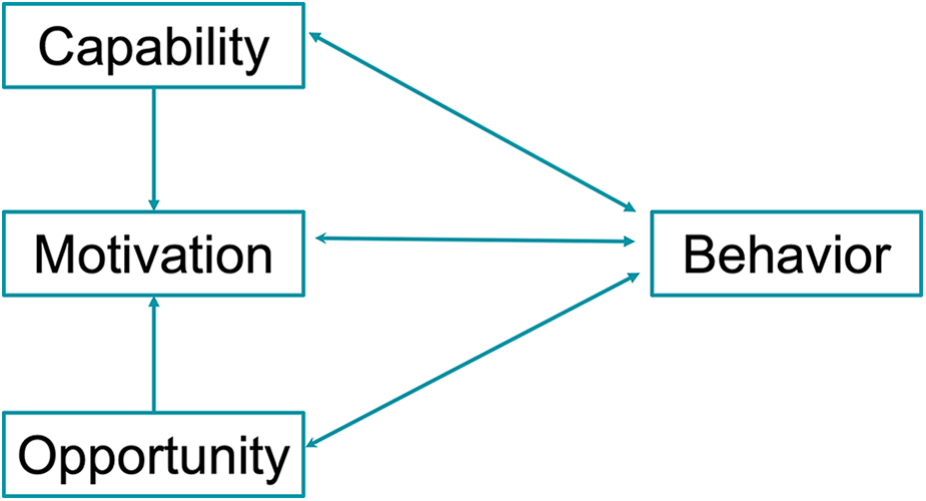
COM-B model (73).

Despite a multitude of existing behavioral change models, their infrequent utilization in designing new interventions led to the development of the Capability, Opportunity, Motivation-Behavior model (COM-B), a new model proposed by Michie et al. (73). In addition to the cognitive elements of traditional behavior theories such as patient beliefs (HBM), reflections (PAPM), motivation (SCM, HAPA), and planning (TPB, HAPA), COM-B also addresses non-cognitive determinants. It hypothesizes that behavior is determined by individual capability, opportunity to perform the action, and motivation to do so. Capability in a dental setting involves both psychological ability (knowledge, comprehension, reasoning, memory, spatial awareness) and physical capacity (manual dexterity, eyesight). Opportunity relates to the external factors that enable and encourage a behavior and may be environmental or social. For dental patients, external opportunities could include having a physical space to perform oral care, such as a bathroom with adequate lighting and a mirror to visualize and evaluate one's performance. Social opportunities stem from cultural or social norms; in Hong Kong, the traditional Chinese medicine belief that “heat” (“yeet-hay”) causes gingival inflammation may direct attention away from the necessity of plaque control (74). Motivation has both an automatic component stemming from impulses, habits and emotions, as well as a reflective component involving planning, decision making and self-reflection. The COM-B model is shown in Figure 4. COM-B has been described as a “behavior system” to emphasize the multidirectional interaction between components of the model and was designed to be used in conjunction with the Behavioral Change Wheel, whereby the COM-B components are linked to intervention functions synthesized from the analysis of multiple theories. It was hoped that the mapping of these functions would assist clinicians in choosing the appropriate technique after understanding which components of behavior need to be addressed, overcoming the difficulty of deciding which theory to apply to a particular situation (75).

As one of the more recent behavior change models, COM-B has been to identify the facilitators and barriers of behavioral change in smoking cessation (76), dietary habits (77) and chlamydia testing (78). Although the COM-B model has been widely described in recent reviews on behavioral change interventions in dentistry (10, 15, 18), its use in clinical settings remains limited (79). Chang et al. (80) tested a mobile application intervention based on COM-B on periodontal patients but did not study whether it let to changes in clinical outcomes. A COM-B based intervention involving behavioral diagnosis and subsequent intervention function selection was tested in athletes (81). At the 12–18 week follow up, a significant increase in oral health knowledge, use of fluoride toothpaste and use of interdental cleaning aids at least 2–3 times per week was noted. However, no effect was noted on bleeding score.

Further study of how the application of the COM-B model impacts periodontal health parameters is needed to gauge its clinical success in dental settings. Such interventions would ideally first diagnose which of capability, opportunity or motivation need to be improved in the patient, then induce behavioral change by targeting the specific components involved. To improve psychological capability, provision of traditional dental education through demonstrations can boost knowledge of oral hygiene techniques and their importance. Physical capability could be managed by referral to a medical practitioner or physiotherapist, or through the provision of specially adapted tools such as an end-tufted single head brush or electric toothbrush. Opportunity could be addressed by planning ways to overcome the existing barriers, such as finding a private restroom at one's workplace to create a physical space to carry out the oral hygiene behavior, creating calendar reminders, or involving social support from family and friends. Motivation can be enhanced by transforming patient beliefs through the use of cognitive behavioral therapy and motivational interviewing Asimakopoulou et al. (10) suggested that the slow uptake of COM-B by dentists lacking a psychology background may be due to assumptions of its complexity. Additional evidence-based examples of its use in oral health interventions would dispel such concerns and provide clinicians with assurance of its viability and effectiveness.

## Summary

The need for patient cooperation in the management of periodontal diseases has long been acknowledged. Growing awareness of the important role that behavior plays in oral health education and the current limited understanding of how theories can be put into clinical practice have led to calls for further studies on theory-based interventions, particularly on recently developed models such as COM-B.

Behavioral change interventions based on several psychological theories were used to improve oral hygiene and periodontal health. Table 1 provides a summary of the recent theory-based intervention studies mentioned in this article that involved an assessment of clinical periodontal outcomes.

**Table 1:** Summary of recent theory-based intervention studies for assessment of clinical periodontal outcomes.

Author (year)	Model/Theory	Design	Intervention	Clinical periodontal variable(s) measured	Follow-up period	Results	Critical analysis
**Interventions based on patient beliefs**							
Sanaeinasab et al. (2022)	HBM	RCT of 112 children in Iran	5 x 1 h educational sessions based on HBM constructs	Papillary bleeding index	3 months	Significant change in papillary bleeding index in intervention group.	Follow-up period could be lengthened. Design could include multiple intervention groups comparing different models.
Xiang et al. (2022)	HBM, SCT	RCT of 1,184 students in Hong Kong	6 x peer-led educational sessions across 6 months (involving leaflets, posters, talks, hands-on workshops, experience sharing, quizzes)	Visible Plaque Index	6, 12 months	Intervention group had larger reduction in visible plaque than control at 12 month follow-up.	Strengths include cluster randomization design with large sample size. As the intervention was also based on SCT, the extent to which the favorable result is due to HBM is unclear.
Rivandi et al. (2018)	HBM	RCT of 196 adult periodontal patients in Iran	3 x individual oral health education using tooth models, 1 x group training session based on HBM constructs	Periodontal probing depths, plaque index with disclosing agent	1, 3 months	Intervention group showed significantly improved plaque index and pocket depths at 1 and 3 months.	No oral health education was provided to the control group, therefore the favorable results may not be due to the model-based education specifically, but the increased time spent with the participants.
Wickremasinghe and Ekanayake, (2017)	HBM	624 students in Sri Lanka	PowerPoint presentation based on HBM constructs, group discussion, toothbrushing demonstration, leaflets	Visible plaque, BOP	6 months	Significant decrease in percentage of sites with plaque and BOP compared to control groups.	Study included two control groups, one which received traditional forms of oral health education not based on HBM followed by a similar group discussion (“didactic” control group), and the other which received only a brief talk (“inactive” control group).
Yekaninejad et al. (2012)	HBM	392 children in Iran	3 x 70 min classroom sessions based on HBM constructs	Simplified oral hygiene index, CPI	3 months	Significantly better oral hygiene compared to control. Gingival health improved, but did not reach significant levels in the intervention group that only involved children. In the comprehensive group, where the intervention involved both students, school staff and parents, significantly improved gingival health was found.	As the subjects are children, their ability to perform oral hygiene may not be as predictable and the full impact of the model-based intervention may not be realized. The inclusion of a test group that involved parents and school staff in addition to the student (“comprehensive” group) overcomes this risk and shows the importance of adult facilitators in child oral health.
Lopez-Jornet et al. (2014)	CBT, MI	RCT of 60 patients with hyposalivation in Spain	4 sessions based on cognitive behavioral principles and motivational interviewing delivered over 2 months.	Plaque extension index, bleeding index, Community periodontal index of treatment needs	2 months	Both intervention and control group (receiving conventional OHI) showed improvement in probing depths, plaque and bleeding indices.	Longer follow-up period would be helpful in assessing longer-term impact of intervention.
Lalic et al. (2012)	CBT, MI	RCT of 99 adolescents undergoing fixed orthodontic treatment	40 min counseling session with elements of MI	Plaque index, gingival inflammation	1, 6 months	Compared to baseline, gingivitis was lower in experimental group. Between groups, no statistically significant differences were observed at 1 and 6 months follow-up.	Baseline motivation was not taken into account when delivering the intervention. The increase in plaque index over time for both groups emphasizes the need for regular, repeated reinforcement.
Jonsson et al. (2010)	CBT, MI	RCT of 113 periodontitis patients in Sweden	Individually tailored oral health education programme based on CBT and MI principles	BOP, periodontal pocket depth, pocket closure, plaque index	3, 12 months	Intervention group had lower BoP and plaque scores at 3 and 12-months. No difference was found for pocket depth reduction.	Strengths include randomized design, large sample and low attrition rates. However, the intervention required specially trained dental hygienists, making the transferability of the programme to general practice more difficult.
**Interventions based on readiness to change**							
Aleksejūnienė and Brukienė (2018)	PAPM	RCT of 166 adolescents in Lithuania	Group-based tailored intervention based on the subject's stage of behavioral change	Digital analysis of photos of stained dental plaque	3, 12 months	Significantly greater improvement in oral hygiene over 12 months in the intervention compared to control.	Oral hygiene assessment was performed at an unannounced visit, so that adolescents could not “prepare” in advance, giving a truer estimate of their general oral health maintenance.
**Interventions based on planning behavioral change**							
Armoon et al. (2021)	TPB	160 hospital staff in Iran	3 x 90 min education sessions (lectures, PowerPoint) tailored to individual needs identified from pre-test questionnaires.	BOP, periodontal screening index	2 months	Significantly lower BOP and PSI in the intervention group. Greater improvement in subjects with greater intention of behavior change as identified in the pre-intervention questionnaire.	Limited follow-up period. PSI has low capacity to discriminate between groups and over time. RCT design was not used.
Wu et al. (2022)	HAPA	RCT of 44 participants with fixed orthodontic appliances in China	Mobile application (“Clean Teeth”) with functions based on HAPA theory, including videos, timer, goal-setting, reminder notifications and achievement recognition.	Gingival bleeding, plaque index with disclosing agent	6, 12 weeks	Significantly lower plaque and gingival bleeding in the intervention group.	Intervention targeted both motivational and volitional phases of the model. Delivery of interventions *via* mobile applications is practical for clinicians and accessible for patients. Follow up period could be lengthened to determine whether improvement was sustained.
Scheerman et al. (2020b)	HAPA	132 adolescents undergoing orthodontic treatment	Mobile application (“WhiteTeeth”) with functions based on HAPA theory, including positive reinforcement, feedback, goal-setting, feedback on photos of disclosing agent use, reminder notifications, coping planning.	Bleeding on marginal probing, plaque index	6, 12 weeks	Significantly greater reduction in dental plaque at 12 week follow up. Bleeding scores improved significantly at 6 weeks, but the significance was lost at 12 weeks.	Comprehensive description of the intervention content was published which aids researchers and clinicians with future implementation planning.
Pakpour et al. (2016)	HAPA	RCT of 1,158 students in Iran	20-minute intervention involving planning the time, location and context of brushing behavior, as well as “if-then” plans.	Visual plaque index, community periodontal index	1, 6 months	Significant difference between intervention and control in both periodontal indices at 6 months.	Although the intervention was delivered by a health psychologist rather than a dentist, the author includes specific instructions to guide its use in clinical practice.
**Interventions based on readiness to change**							
Suresh et al. (2012)	SRT	Prospective trial of 73 periodontal diseaes patients	Self monitoring *via* diary flossing calendar.	Interproximal plaque and bleeding scores of 6 reference teeth	4 weeks	Reduced plaque and bleeding scores were noted regardless of initial readiness to change, assessed by pre-intervention questionnaires.	Short follow-up period does not allow for lasting changes to be evident. Study did not involve a control group and relied on self-report measures to assess readiness to change.

BOP, bleeding on probing; CBT, cognitive behavioral therapy; HAPA, health action process approach model; HBM, health belief model; MI, motivational interviewing; PAPM, precaution adoption process model; RCT, randomized controlled trial; SCM, stages of change model; SCT, social cognitive theory; SRT, self-regulation theory; TPB, theory of planned behavior.

It is difficult to draw direct comparisons between interventions due to heterogeneity in constructs, methodology, strength of evidence and standards of reporting. The extent to which each intervention was based on its underlying behavioral change theory could not be accurately determined, with some interventions incorporating aspects from multiple theories (27, 38, 82). This ambiguity presents difficulties in determining whether specific components of the interventions are more clinically effective than others. There is greater need for researchers to clearly describe the interventions employed from each model and adopt a common framework to define and assess interventions. In addition, the observation period for different studies varies significantly and the longest follow-up period was 12 months, hence the short- and long-term clinical impact of the different psychological theories could not be compared and assessed. Moreover, as several of the theories require discussion of patient beliefs and goals, it is unclear whether the observed clinical improvements partially result from the additional time spent with the patients, rather than the underlying theoretical principles. Finally, the psychological models included in this article are not exhaustive and the reader is directed to relevant reviews for further understanding of additional models that have been used to guide dental interventions (3, 15, 16, 18).

Despite the theoretical and methodological differences between the interventions, a commonality they all share is the use of an evidence-based theoretic framework to understand the constructs of behavior and the development of measures to evaluate these constructs. Compared to conventional dental education, theory-based interventions consider the patient's beliefs, cognition, self-efficacy, level of readiness and socio-environmental context in the delivery of oral health instruction (83). This results in an individually tailored, person-centered approach with a mutual understanding of the patient's specific goals and challenges. Dental professionals must build rapport with their patients to achieve the close relationship necessary to support behavior change. While it is the patient himself who is empowered to take control and responsibility for initiating and maintaining the behavior, the dentist has the responsibility of eliciting information about the patient's behavioral determinants and guiding the intervention. It is therefore imperative that dentists, as healthcare providers, understand the complexity of behavioral change and are educated on evidence-based OHIs to achieve holistic and sustainable treatment outcomes. The grouping of behavioral change interventions in this article underscores the components of individual behavior and the key concepts of behavior change theory that a clinician must consider when developing interventions. To facilitate the development of a tailored, patient-centered oral hygiene intervention, the stage of readiness to change must be assessed and individual beliefs, motivation, capability explored. This can be achieved through cognitive behavioral interventions, goal-setting, or theory-driven education. On a practical level, barriers to action can be identified and the creation of opportunities elicited through planning and environmental change.
